# Test your knowledge and understanding

**Published:** 2017-08-07

**This page is designed to help you to test your own understanding of the concepts covered in this issue, and to reflect on what you have learnt.**

**Figure:**
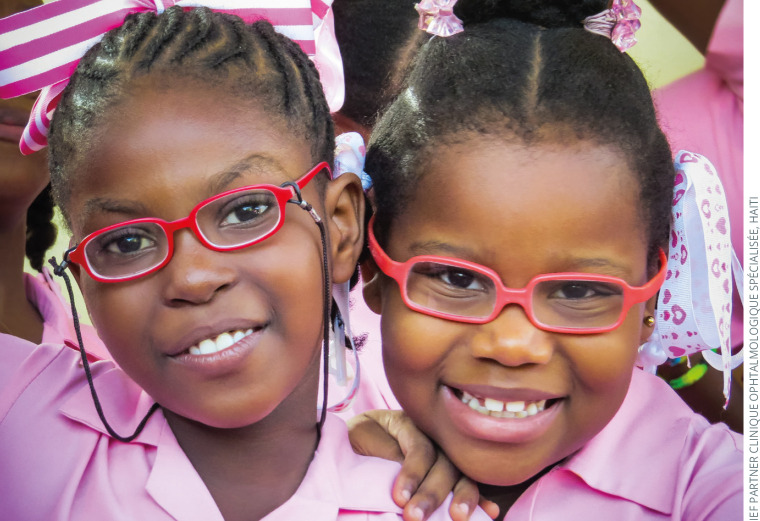
New spectacles make all the difference for two young school girls. HAITI

We hope that you will also discuss the questions with your colleagues and other members of the eye care team, perhaps in a journal club. To complete the activities online – and get instant feedback – please visit **www.cehjournal.org**

**Tick ALL that are TRUE**

**Question 1**
**The following children, seen by a school teacher, need referral to an eye trained health worker:**
  □ **a**. Child with a red eye
  □ **b**. Child with convergent squint
  □ **c**. Child with 6/18 vision in both eyes
  □ **d**. Child with a white pupil in one eye
  □ **e**. Child with 6/6 and 6/9 vision
**Question 2**
**The following are reasons why children may not wear prescribed spectacles:**
  □ **a**. Too expensive
  □ **b**. Do not fit properly
  □ **c**. Teased by other children
  □ **d**. Parents do not think they are important
  □ **e**. Cannot see any better
**Question 3**
**The following are important indicators to monitor a school screening programme for refractive error:**
  □ **a**. Total number of children in the school and the number who had their vision screened
  □ **b**. Number of children who failed the visual acuity test
  □ **c**. Number of children who were refracted
  □ **d**. Number of children who had spectacles prescribed
  □ **e**. Number of children who are using spectacles 3–6 months after the spectacles were prescribed
**Question 4**
**School eye health programmes need to:**
  □ **a**. Have the approval of the ministry of education
  □ **b**. Be funded through the sale of children's spectacles
  □ **c**. Be done once for all schools in a district every 5–10 years
  □ **d**. Be part of a broader school health programme
  □ **e**. Include eye health for teachers

## ANSWERS

1. a, b, c and d are correct. A white pupil (leukocoria) may be due to a cataract or other serious eye condition. It is unlikely that the child in (e) will need (or wear) spectacles.
2. All are correct.
3. All are correct.
4. a, d and e are correct. The programme should ideally be funded by the ministry of education or other institutional donors, not from sale of spectacles to children (which can limit uptake). The screening should occur in each school every 1–2 years, when new students are screened and those who failed the test in previous years are re-examined; every 5–10 years is too infrequent.

